# Hemocompatibility of new magnetically-levitated centrifugal pump technology compared to the CentriMag adult pump

**DOI:** 10.1038/s41598-020-78709-0

**Published:** 2020-12-16

**Authors:** David Schibilsky, Setsuo Takatani, Barbara Schibilsky, Tobias Graf, Diana Michels da Silva, Hans Peter Wendel, Meltem Avci-Adali, Christian Schlensak

**Affiliations:** 1 Department of Cardiovascular Surgery, University Heart Center Freiburg Bad Krozingen, Freiburg, Germany; 2 Tokyo Medical and Dental University, Tokyo, Japan; 3 Department of Cardiology, Angiology and Intensive Care Medicine, University Medical Center Schleswig-Holstein, Campus Luebeck, Luebeck, Germany; 4 Department of Thoracic and Cardiovascular Surgery, University Medical Center Tuebingen, Hoppe-Seyler Str. 3, 72076 Tübingen, Germany

**Keywords:** Experimental models of disease, Translational research

## Abstract

The specific hemocompatibility properties of mechanical-circulatory-support (MCS)-pump technologies have not previously been described in a comparable manner. We thus investigated the hemocompatibility-indicating marker of a new magnetically-levitated (MagLev) centrifugal pump (MT-Mag) in a human, whole-blood mock-loop for 360 min using the MCS devices as a driving component. We compared those results with the CentriMag adult (C-Mag) device under the same conditions according to ISO10993-4. Blood samples were analyzed via enzyme-linked-immunosorbent-assay (ELISA) for markers of coagulation, complement system, and the inflammatory response. The time-dependent activation of the coagulation system was measured by detecting thrombin-anti-thrombin complexes (TAT). The activation of the complement system was determined by increased SC5b-9 levels in both groups. A significant activation of neutrophils (PMN-elastase) was detected within the C-Mag group, but not in the MT-Mag group. However, the amount of PMN-elastase at 360 min did not differ significantly between groups. The activation of the complement and coagulation system was found to be significantly time-dependent in both devices. However, coagulation activation as determined by the TAT level was lower in the MT-Mag group than in the C-Mag group. This slight disparity could have been achieved by the optimized secondary flow paths and surface coating, which reduces the interaction of the surface with blood.

## Introduction

New technologies and growing clinical experience have led to a dramatic increase in the use of mechanical circulatory support systems and to the improvement of outcomes after short and long-term support^1,2^. However, complications associated with the interaction of blood with foreign surfaces remain. Thrombosis and hemolysis caused by long-term mechanical circulatory support (MCS) devices are associated with worse outcome and severe morbidity^3^.

As the interventional techniques in cardiovascular medicine continue to develop and ever more critically-ill patient cohorts undergo treatment, the demand for mechanical circulatory support is increasing dramatically in this field. Its prophylactic and bail-out application for percutaneous mitral valve repair and TAVI has been reported with satisfactory outcomes^4,5^. The hemocompatible properties of rotary blood pumps commonly used in MCS have not been adequately described. Most investigations of different pump technologies focus on hydrodynamic properties and hemolysis^6,7^. Although there are some theories about differently distributed shear stress between different pump technologies, there is no evidence of the main advantage of one pump over another^8^.

Most currently available extracorporeal MCS pumps rely on blood immersed-bearing technology (e.g. BioPump BPX-80, Medtronic, Minneapolis USA Rotaflow and CardioHelp, Maquet, Germany; Deltastream, Medos, Germany). Bearings immersed in blood share the potential risk of thrombus formation in the bearing area^9–11^. To reduce this potential risk of such pumps, magnetic levitation technologies have been proposed that offer a lower risk of thrombosis and more durable performance for the mid-term support of extracorporeal circulation.Most experimental models aiming for a detailed biocompatibility analysis include an oxygenator. Therefore, those investigations pay less attention to the impact of the pump technology itself^12,13^. The individual hemocompatibility properties of various pump technologies have not been compared in the same experimental model.

The new mag-lev centrifugal pump technology, MT-Mag, was developed by the Japanese company MedTech Heart, Inc., Tokyo Japan. The hydraulic and hemolytic properties of the new pump had recently been tested^14^. Since the hemocompatible properties had not yet been evaluated or even compared to other pump technologies, we decided to analyze the performance of the new MT-Mag pump within a whole blood mock loop and compared it to the best clinical standard, namely the magnetically levitated centrifugal CentriMag (C-Mag). For this purpose, we analyzed the hemocompatible performance of the MT-Mag versus the C-Mag’s under identical experimental conditions according to ISO10993-4.

## Materials and methods

### Comparison of MT-Mag and C-Mag characteristics

**Figure 1:**
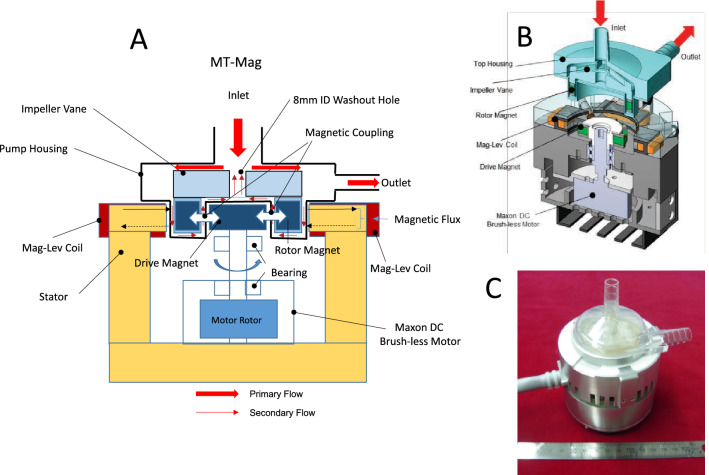
**(A)** Schematic drawing of the MT-Mag pump head inserted in the motor drive, **(B)** CAD design of the MT-Mag, C: Assembled MT-Mag prototype.

The MT-Mag and C-Mag are magnetically levitated centrifugal blood pumps^6,12,14^. The design features of the MT-Mag are shown in Fig. 1 with its characteristics summarized in Table 1. The MT-Mag is a bearing-free mag-lev centrifugal pump (MedTech Heart Inc., Tokyo, Japan) that has not yet received a CE-Mark. The C-Mag, originally developed by Levitronix (Zurich, Switzerland) and sold to Thoratec (Thoratec Ltd., Pleasanton, USA) in 2012, was certified for 30-day use by CE-Mark in 2009. The C-Mag also acquired premarket notification or 510 K* in the United States (*Premarket notification or known as 510 K is the process for placing a new device on the market whose safety and effectiveness are similar to the predicate devices on the market). The C-Mag is currently undergoing clinical trials to obtain premarket approval (PMA) in the United States with IDE (investigational device exemption) approved in 2013.The centrifugal-type of 6 impeller vanes of the C-Mag and MT-Mag, which are mounted on the top of the rotor, is comparable. Both pumps draw blood from a 3/8-in. inner diameter inlet port and expel the blood through a 3/8-in. outlet port. C-Mag’s rotor is disc-shaped, while that of the MT-Mag is shell-shaped. However, the external diameter is within the same range of about 50 mm. The MT-Mag’s priming volume including inflow and outflow ports is 23 ml and therefore slightly smaller than the C-Mag’s 31 ml priming.The washout hole with an inner diameter of 8 mm is located at the center of the impeller with the secondary flow channel of 0.3 mm width in the MT-Mag. This allows the blood to recirculate inside the pump head from the outlet port to the inlet port area. The recirculated flow merges with the incoming flow at the inlet port. The computational fluid-dynamic analysis showed that about 10% of the external flow recirculates through this secondary flow channel^14^. The C-Mag pump also has a central washout hole to recirculate flow from the outflow port to the central inlet port area, however, the amount of recirculating blood has never been measured or published. Due to the larger gaps of around 1 mm within the C-Mag, we can assume that its recirculating volume is higher. This argument is reinforced by the fact, that the C-mag needs higher pump speed than the MT-Mag to ensure the same pump flow.

**Table 1:** Technical specifications of the MT-Mag pump.

Levitation MT-Mag	Magnetic levitation with 2-axis (X- and Y-axis) active control Z- and tilting axis passive control with a strong permanent magnet Z-axis stiffness = 57.6 N/mm^15^
Drive (impeller rotation)	Magnetic coupling drive
Priming volume (ml)	23
Wash-out hole	8 mm ID central wash-out hole with 0.3 mm secondary flow channel
Max RPMs	5000
Max flow L/min	10
Maintenance capability	Off-the-shelf motors allow easy repair with low cost
Monitoring capability	Pump rpm/flow, blood pressures (3 channels), Hb oxygen saturation and Hb content
Surface coating	MPC polymer coating
Indication	ECMO, ECLS, VAD (LVAD, RVAD, BiVAD),
Production cost	Less expensive

*VAD* ventricular assist device, *LVAD* left ventricular assist device, *RVAD* right ventricular assist device, *BiVAD* bi-ventricular assist device, *ECMO* extracorporeal membrane oxygenation, *ECLS* extracororeal life support, *MPC* 2-methacryloyloxyethyl phosphorylcholine.

The major design difference in levitation or floating with magnetic field and drive mechanism between the MT-Mag and C-Mag: The MT-Mag utilizes an off-the-shelf motor to rotate the floating impeller, while the C-Mag integrates the impeller rotor within the motor and both levitation(X-, Y- and Z-axis), and impeller rotation is triggered through magnetic induction to enable a bearing-less Mag-Lev pump^14^. The MT-Mag, on the other hand, actively controls X- and Y-axis impeller displacement using two sets of electromagnets. While the Z- and tilting-axis are passively controlled using the restoring forces developed between the driver magnet and impeller rotor magnet, the impeller rotation is induced via radial magnetic coupling between the outer follower magnet inside the impeller rotor and a strong inner driver magnet mounted on the shaft of a commercially-available brushless direct current (DC) motor (Maxon, Inc., Swiss). Due to the strong magnetic coupling force between the impeller rotor and driver magnet, extremely stiff levitation with Z-axis stiffness measuring 57.6 N/mm. was demonstrated. The Z-axis stiffness of the C-Mag measures approx. 2.7 N/mm. This experimental evaluation reveals that a stiffness value of 57.6 N/mm can withstand external disturbances equivalent to 15G acceleration force^15^.

The MT-Mag’s blood-contacting surface contains a 2-methacryloyloxyethyl phosphorylcholine (MPC) polymer coating, whereas the C-Mag’s surface is not coated. This MPC polymer coating suppresses the adhesion of blood proteins to the surface by mimicking cell-membrane characteristics, thus preventing the adhesion of blood cells and consequently thrombus formation on the foreign surface^16^. Both pumps exhibit similar flow characteristics with maximum pump speed ranges from 0 to 5000 rpm and a maximum flow capability of 10 L/min^12,14,15^.

### Preparation of mock loop circuits

We investigated the MT-Mag device and C-Mag adult pump in a fresh human whole-blood mock loop circuit. Thus 500 ml of fresh human whole blood was circulated for 360 min in a closed-loop model using the MCS devices as driving components (C-Mag n = 3; MT-Mag n = 5). Each of the 8 tests was performed with new pumps and circuits with new fresh human whole blood (500 ml) from an individual donor (in total: 8 donors). The human blood was provided by the University Hospital blood donation service according to university guidelines. Blood was collected from healthy donors, which gave written informed consent to participate. The blood sampling procedure was approved by the Ethics Committee of the Medical Faculty of the University of Tuebingen. The period from blood donation to the start of experiments was under 30 min. 500 ml of fresh blood (3 IU Heparin/ml blood [ratiopharm, Ulm, Germany]) was put in a blood bag serving as a reservoir for a closed circuit tubing mock loop. The tubing consists of 3/8 non-coated standard extracorporeal circulation tubes (ECC noDOP 3/8" × 3/32", Rehau, Germany). The MCS pump (MT-Mag’s or C-Mag’s) was incorporated as a driving component at the circuit’s deepest level. Since these circuits contain no oxygenator, there was no chance of heating. The initial temperature of the human blood was 37 °C.

### Experimental setup

In the C-Mag group, a blood flow of 5.0 L/min was maintained by running the C-Mag pump at a rotational speed around 2200 rpm. In the MT-Mag group, a rotational speed of 1800 rpm resulted in a blood flow of 5.0 L/min. Blood samples were taken from the arterial site of the perfusion circuit at 0, 1, 5, 10, 20, 30, 60, 120, 180, 270 and 360 min after starting the circulation.

### Analysis

The cell counts were analyzed immediately after collecting the blood into EDTA-containing tubes using an automated cell counter (Sysmex K 1000, Sysmex Corporation, Kobe, Japan). Changes in markers of coagulation, complement activation, and blood cell release factors were measured via enzyme-linked immunosorbent assay (ELISA). Samples were analyzed for coagulation marker thrombin-antithrombin III (TAT), polymorphonuclear-elastase (PMN-elastase) and terminal complement complex SC5b-9. The ELISA-kits (SC5b-9: Quidel, San Diego, CA, USA, TAT: Dade-Behring, Marburg, Germany; PMN-elastase: Boehringer, Mannheim, Germany) were used according to the manufacturer's instructions.

### Data analysis

Data were analyzed using GraphPad Prism (Version 6.0c, GraphPad Prism Software, Inc.; La Jolla, CA). Our results are presented as mean ± SEM. Normal distribution was present in previous experiments using the same experimental setup, but could not be verified, as the numbers were too low^17^. Therefore, we used the unpaired *t*-test to compare ranks. A p-value of ≤ 0.05 was considered as statistically significant.

## Results

### Blood flow and device setting

The blood flow of the C-Mag group was maintained at 5.0 ± 0.2 L/min, and in the MT-Mag group, the rotation speed of the MT-Mag was set to 1650–1770 rpm, resulting in a flow of 5.0 ± 0.2 L/min, while the rotation speed of the C-Mag was higher, ranging from 2300 to 2500 rpm.

### Cell count and hemolysis

**Figure 2:**
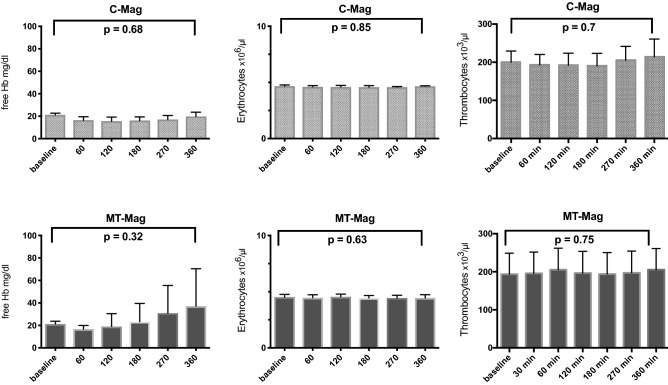
Plasma-free hemoglobin, as well as cell counts of erythrocytes and thrombocytes in the MT-Mag and C-Mag groups. Hemolysis and numbers of erythrocytes and thrombocytes were stable in both groups throughout the experimental course.

Erythrocyte counts were stable within the C-Mag and MT-Mag groups throughout the 360 min of our experiment (C-Mag: 4.6 ± 0.06 × 10^6^ /µl vs. 4.6 ± 0.02 × 10^6^ /µl, p = 0.85; MT-Mag: 4.5 ± 0.1 × 10^6^ /µl vs. 4.4 ± 0.1 × 10^6^ /µl, p = 0.63) (Fig. 2). The number of thrombocytes also remained stable in both groups (C-Mag: 200.0 ± 16.9 × 10^3^ /µl vs. 214.2 ± 33.0 × 10^3^ /µl, p = 0.7; MT-Mag: 193.6 ± 24.7 × 10^3^ /µl vs. 205.4 ± 24.9 × 10^3^ /µl, p = 0.75) (Fig. 2). The MT-Mag group’s free hemoglobin level revealed a tendency to increase, but failed to reach significance in either group (C-Mag: 21.1 ± 0.9 vs. 19.7 ± 2.3 mg/dl, p = 0.68; MT-Mag: 21.3 ± 1.1 vs. 37.12 ± 14.9 mg/dl, p = 0.32) (Fig. 2).

### Coagulation

The TAT plasma concentration, an excellent marker for detecting coagulation activation, was measured immunochemically via the ELISA method. Thrombin formation results in prothrombin fragment F 1 + 2 release from prothrombin. The remaining thrombin is inactivated by complex-formation with antithrombin-III, which leads to the TAT complex.

**Figure 3:**
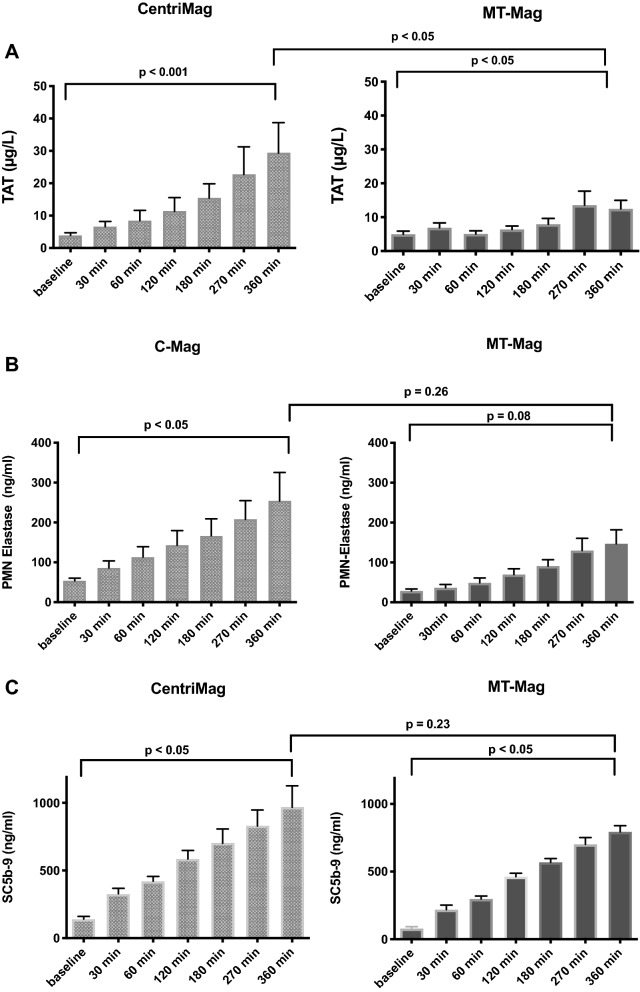
**(A)** ELISA quantification of thrombin–antithrombin III complex (TAT) as a marker of coagulation activation. Both groups revealed a significant increase of up to 360 min during the experiment. TAT values were higher in the C-Mag group than the MT-Mag group (29.4 ± 5.4 µg/L vs. 12.5 ± 2.5 µg/L, p < 0.05). **(B)** ELISA quantification of blood levels of polymorphonuclear (PMN)-elastase as an inflammation marker. C-Mag and MT-Mag group PMN values were comparable (162.2 ± 58.2 ng/mL vs. 253.8 ± 71.5 ng/mL, p < 0.26) after 360 min. **(C)** Blood levels of SC5b-9 as a key element within the complement cascade detected by ELISA. After 360 min both groups ‘ values were comparable (967.9 ± 158.2 ng/mL vs. 793.2 ± 74.4 ng/mL ng/mL, p = 0.23).

The increase in TAT from baseline to 360 min was statistically significant within the C-Mag (3.9 ± 0.5 µg/L vs. 29.4 ± 5.4 µg/L, p < 0.001) and MT-Mag group (5.0 ± 2.1 µg/L vs. 12.5 ± 2.5 µg/L, p < 0.05) (Fig. 3A). At 360 min, the C-Mag group’s TAT values were higher than the MT-Mag group’s (29.4 ± 5.4 µg/L vs. 12.5 ± 2.5 µg/L, p < 0.05). This might reflect the MT-Mag group’s weaker coagulation activation (Fig. 3A).

### Inflammation

The amount of PMN-elastase was quantified by ELISA. Activated PMN granulocytes lead to the expression of membrane proteins and the release of elastase and other substances out of their granules. Unbound elastase is complexed with the alpha1-proteinase inhibitor. Thereby, the amount of PMN-elastase complex is a quantitative marker of inflammation.

PMN-elastase values increased significantly from baseline until the end of the experiments (360 min) in the C-Mag group (53.4 ± 6.6 ng/mL vs. 253.8 ± 71.6 ng/mL, p < 0.05), but not in the MT-Mag group (27.1 ± 6.9 ng/mL vs. 162.2 ± 58.2 ng/mL, p = 0.08) (Fig. 3C). However, at the end of the experiment, the MT-Mag’s and C-Mag adult groups’ values were comparable 162.2 ± 58.2 ng/mL vs. 253.8 ± 71.5 ng/mL, p = 0.26) (Fig. 3B).

### Complement system

The end product of the complement system SC5b-9 [also called membrane attack complex (MAC)] is generated via complement factor C3a. The amount of SC5b-9 is therefore a quantitative marker of complement activation and can be measured via ELISA.

SC5b-9 values increased significantly from baseline to 360 min within both the C-Mag (141.8 ± 19.1 ng/mL vs. 967.9 ± 158.2 ng/mL, p < 0.05) and MT-Mag group (62.9 ± 18.0 ng/mL vs. 793.2 ± 74.4 ng/mL, p < 0.05) (Fig. 3B). The group difference in values at 360 min did not reach significance (967.9 ± 158.2 ng/mL vs. 793.2 ± 74.4 ng/mL, p = 0.23) (Fig. 3C).

## Discussion

To the best of our knowledge, this is the first study to compare the hemocompatibility of two magnetically levitated centrifugal pump technologies of different designs with an identical experimental setup using human blood. The newly developed, magnetically-levitated MT-Mag pump exhibited a hemocompatibility similar to the CentriMag (C-Mag) device, which is the clinical gold standard. The MT-Mag even demonstrated a weaker activation of the coagulation system than the C-Mag. The high level of hemocompatibility of the new pump, which is similar to the gold standard, is most likely due to the similar magnetic levitation technology in both pumps. The potential advantage of the newly designed MT-Mag pump could lie in differences between the design and surface characteristics of the two pump technologies.

Concerning the basic pump design, the shell-shaped MT-Mag impeller accompanied by the central wash-out hole leads to lower recirculation flow through secondary paths compared to the C-Mag. The recirculation flow is directed from the outlet port area to the inlet port area through the 0.3 mm gap between the pump housing and impeller rotor in the MT-Mag. A computational fluid dynamics study of the MT-Mag revealed that approximately 10% of the external flow recirculates through the secondary flow channel^15^. These optimized flows, together with the surface coating (non-active phosphorylcholine polymer), might help to prevent blood stagnation inside the pump, suppress activation of the coagulation system and thereby results in the generation of less TAT. The C-Mag pump also has a central washout hole to recirculate flow from the outflow port to the central inlet port area. However, the actual amount of recirculating blood has never been measured or published. Since the gaps within the C-Mag are considerably larger (1 mm), we can assume that the recirculation volume could be higher compared to the MT-Mag. This assumption is supported by the fact that the C-Mag required a higher pump speed possible due to the greater recirculation flow inside the pump reducing pump efficiency. This might lead to greater shear stress within the pump and could explain the increased activation of the coagulation system.

Furthermore, pump technologies without recirculation paths can cause blood to become trapped in the bottom space between the impeller bottom and pump housing. By creating spiral flow patterns in this region, blood is retained for too long, which potentially causing an enhanced activation of the coagulation system^12^. The MT-Mag utilizes MPC surface coating. Thus, this coating of the pump-head might also contribute to hemocompatibility. The phosphorylcholine polymer MPC mimics the cell membrane to suppress the adhesion of blood proteins. It does not actively interact with the blood, but passively suppresses the adhesion of the blood proteins to the blood-contacting surface^16^. Thus, the higher TAT values of the C-Mag group after 360 min could be partly explained by their lack of a hemocompatible surface coating. The contact of platelets and other blood cells with foreign materials can lead to activation and result in thrombin generation, which in turn can bind to circulating anti-thrombin complex III to form thrombin-anti-thrombin III complex (TAT). TAT levels might also be enhanced by the adhesion of blood proteins to the uncoated surface. The C-Mag demonstrated acceptable hemocompatibility and adverse event-rate profile in patients^18^. However, the persistent high rates of adverse events associated with extracorporeal life support (ECLS)/extracorporeal membrane oxygenation (ECMO) therapy generally indicate the need to develop techniques that reduce surface interactions within both pump technologies^1^.

Hemocompatibility studies of new MCS devices are often conducted with a whole system including an oxygenator^19^. Therefore, their data cannot be used to compare the hemocompatibility of different pump designs used a circuit without an oxygenator. The same limitation applies to investigations of the hemocompatibility profile of the C-Mag adult pump in an ECMO setting^13^. Most other studies focused on hydrodynamic properties and hemolysis of new pump systems. Nevertheless, these investigations do not provide specific data on device-blood interactions^20,21^. In our previous study, the C-Mag device revealed significantly less coagulation and inflammation activation than the HeartMate II axial flow pump^22^, reflecting the improved hemocompatibility of the magnetically-levitated pumps. The investigation of other magnetically levitated centrifugal pump designs such as the new MT-Mag system in the same experimental setup is of great interest. A biocompatible surface coating could effectively reduce MSC-associated hemostatic activation cascades^17^. The ultimate goal in designing future blood-contacting surfaces will be the active ability of MCS system to endothelialize in vivo to substantially improve biocompatibility^23^.

### Study limitations

In this in vitro study, the MT-Mag design in combination with a secondary flow path and an MPC-polymer surface coating revealed promising differences in activation of coagulation system. Blood from healthy volunteers was used in these experiments. Their blood is obviously not identical to the blood of patients with advanced heart failure (HF). Nevertheless, some components of the hemostatic system like platelet or coagulation activation are even more sensitive in healthy people than in HF patients. It was therefore appropriate to use blood from healthy volunteers in this first preclinical study of a new type of pump. Since the markers (TAT, PMN-elastase, and SC5b-9) were not detected during a clinical treatment of patients, the clinical relevance of these findings will have to be proven in clinical investigations. Our study provides short-term in vitro data with small sample numbers. However, our statistical calculations prove the safety of a new device in comparison to a clinically well-established pump. The relevance of our findings in terms of mid-term in vivo applications in patients thus requires further investigation.

## Conclusion

In this study, using an in vitro mock-loop setting, we demonstrated a time-dependent activation of the coagulation and complement system via the C-Mag and MT-Mag device. Their hemocompatibility profiles are comparable. However, the MT-Mag device revealed a significantly lower coagulation activation than the CentriMag adult. These results suggest that both magnetically levitated pump systems possess a good hemocompatibility profile. The surface coating of the MT-Mag and optimized secondary flow characteristics could have given a slight advantage in preventing coagulation activation. However, these results need to be proven by mid-term in vivo investigations.
